# Statics Performance and Heat Dissipation Evaluation of Lattice Structures Prepared by Laser Powder Bed Fusion

**DOI:** 10.3390/mi15070888

**Published:** 2024-07-07

**Authors:** Jingfei Bai, Can Zhang, Ziche Li, Ruobing Liao, Zhengxing Men, Liang Wang, Chen Ji, Kun Li

**Affiliations:** 1 Chengdu Aeronautic Polytechnic, Chengdu 610100, China; 2 Aerospace Life-Support Industries, Ltd., Xiangyang 441003, China; 3 College of Mechanical and Vehicle Engineering, Chongqing University, Chongqing 400044, China20220701068@cqu.edu.cn (C.J.); 4 Chongqing Key Laboratory of Metal Additive Manufacturing (3D Printing), Chongqing University, Chongqing 400044, China

**Keywords:** laser, selective laser melting, porous aluminum magnesium alloy, support structure

## Abstract

This paper address the performance optimization of the battery heat sink module by analyzing the lattice structure of the battery heat sink module through in-depth modeling and simulation, and combining the laser powder bed fusion (LPBF)-forming technology with mechanical and corrosion resistance experiments for a comprehensive study. It is found that the introduction of the lattice skeleton significantly improves the thermal conductivity of the phase change material (PCM), realizing the efficient distribution and fast transfer of heat in the system. At the same time, the lattice skeleton makes the heat distribution in the heat exchanger more uniform, improves the utilization rate of the PCM, and helps to maintain the stability of the cell temperature. In addition, the melting of PCM in the lattice heat exchanger is more uniform, thus maximizing its latent heat capacity. In summary, by optimizing the lattice structure and introducing the lattice skeleton, this study successfully improves the performance of the battery heat dissipation system, which provides a strong guarantee for the high efficiency and stable operation of the battery, and provides new ideas and references for the development of the battery heat dissipation technology.

## 1. Introduction

Heat exchangers are used in many fields, from home appliances such as refrigerators, air conditioners, etc., to the vigorously developed electric vehicle field, and even aerospace [1,2]. With the increasing maturity of 3D printing technology, combining 3D printing with heat exchangers has become a focus of current research because through 3D printing, the design of lightweight heat exchangers can be reduced, unnecessary material waste can be reduced, energy consumption can be reduced, and the heat transfer capacity of heat exchangers can be enhanced. In addition, 3D printing can use a variety of materials, including metals, plastics, ceramics, etc., to adapt to different heat transfer needs. For example, metal 3D printing can be used to manufacture high-temperature and high-pressure heat exchangers, while polymer 3D printing can be used for low-temperature applications. Therefore, the application prospect of 3D printing technology in the field of heat exchangers is very broad. Heat exchange manufactured with 3D printing manufacturing technology has strong potential in terms of manufacturing time and performance characteristics. Romei et al. [3] introduced the design, manufacture, and post-production analysis of a new high-temperature resistance jet heat exchanger for spacecraft, and verified the feasibility of this manufacturing method, opening up new ideas for the development of multiple industries in the future. Dixit et al. [4] designed a microstructured gyro lattice liquid–liquid compact heat exchanger, which was manufactured by 3D printing technology. This compact heat exchanger is light in weight and has a porosity of 80%. The finite element analysis results show that the heat exchange efficiency of this heat exchanger is increased by 55% compared with the thermodynamically equivalent and most efficient countercurrent heat exchanger. The heat exchanger is only one-tenth the size of the former. Saltzman et al. [5] compared the performance of traditional plate-fin air–liquid cross-flow heat exchangers (i.e., aircraft oil coolers) with similar geometric shapes, and the research results showed that the total heat transfer of the heat exchanger manufactured by additive was increased by 14% compared with traditional heat exchangers. The porous metal structure can significantly reduce the weight of the heat sink, and the common structures include the metal foam with irregular holes, the metal lattice structure with regular holes, and the three-period minimum surface (TPMS) structure of complex holes. Zhou et al. [6] studied the melting process of 3D-printed metal foam composite PCM, and the results showed that adding 3D-printed ALSI10MG aluminum alloy foam metal with a porosity of 0.838 could significantly increase the melting speed of the composite PCM by 2.5 times, and the temperature field of the composite PCM was more uniform. Grande et al. [7] studied the influence of 3D-printed aluminum lattice on the thermal effect of rapid adsorption of methane on HKUST-1, and the research results showed that the use of internal 3D-printed aluminum lattice significantly reduced the temperature when the tank of HKUST-1 was quickly filled with methane. Ramirez et al. [8] used 3D printing technology to prepare copper mesh and open-cell foam materials, and these open cellular structural components have shown considerable potential for novel, complex, multifunctional electrical and thermal management systems, especially complex monolithic heat exchange devices. Fratalocchi et al. [9] used a 3D-printed periodic open-hole structure to promote heat exchange in a fixed-bed FT reactor, and the results showed that the 3D-printed open-hole metal enhanced heat transfer by improving the radial thermal conductivity between the scaffold and the reactor wall. Sun et al. [10] used additive manufacturing technology to manufacture heat sinks, and verified the performance of heat sinks through experimental tests. The results showed that the thermal conductivity was doubled compared with the traditional rectangular fin heat sinks. Some scholars have studied the heat transfer performance of heat exchangers based on three-period minimum surfaces (TPMSs). TPMSs are difficult to obtain through traditional processing due to their complex structure, while different types of TPMSs can be processed through 3D printing. Qian et al. [11] successfully manufactured three kinds of high-thermal-conductivity pure copper TPMS heat exchangers with different structures by using additive manufacturing technology. Compared with traditional heat exchangers, the heat exchange efficiency of these three heat exchangers has been improved to varying degrees. Reynolds et al. [12] studied these different types of TPMSs, and the research results showed that the helical dodecahedral TPMS design had high heat transfer rate and moderate pressure drop. Compared with the straight tube heat exchanger, the Nusser number of helical dodecahedral TPMS increased by 13%. In the heat transfer of fluid boundary, Nusser number represents the ratio of convective heat across the boundary to conducted heat, and the larger the Nusser number is, the larger the convective heat transfer coefficient is. Samson et al. [13] found that TPMSs are rapidly becoming a heat sink and heat exchanger topology superior to traditional designs and other honeycomb structures. For the combination of TPMSs and air cooling to form a battery thermal management system, TPMSs can make the cooling air flow through the formation of more spiral flow, strengthen the heat exchange efficiency, and the use of the radiator Nussel number compared with the traditional fin radiator increased by 300%. Lu et al. [14] proposed using digital light processing (DLP) printing technology to construct a three-period minimum surface (TPMS) skeleton, and impregnated h-BN on the surface of the skeleton to improve the overall thermal conductivity of the composite. When the addition of h-BN was 20 vol%, the thermal conductivity of the composite was 1.86 W/(m·K). The thermal conductivity is 786% higher than that of pure epoxy resin.

In addition, 3D printing technology can be used to manufacture complex radiators and cooling channel structures, which may be difficult to achieve using traditional manufacturing methods. These complex structures can optimize heat exchanger performance, improve heat transfer efficiency to help control equipment temperature, and extend service life. Gu et al. [15] used 3D printing technology to design and manufacture a flat plate heat exchanger with an aluminum-based lattice structure. The experimental results showed that under the condition of high heat load, the maximum temperature of the heat exchanger with 3D-printed lattice structure was 55.5 °C, 19.46% lower than that of the solid structure, and the maximum temperature difference was only 4.51 °C when the heat load was 120 w, which fully proves that the addition of the 3D-printed lattice structure can improve the overall heat transfer capacity of the heat exchanger. Das et al. [16] developed a design for a new countercurrent pin-fin compact heat exchanger, manufactured using 3D printing technology. The heat exchanger can operate at high temperatures of 800 °C and withstand a fluid pressure difference of 170 bar with a design life of 40,000 h. Ahmadi et al. [17] designed a high-thermal-conductivity polymer bionic lung heat exchanger by 3D printing. The research results show that the efficiency of the high thermal conductivity polymer heat exchanger made by 3D printing is comparable to that of the metal heat exchanger made of stainless steel and aluminum, and the pressure drop of the coolant of the heat exchanger is only one-quarter of that of the common metal plate heat exchanger, significantly reducing energy consumption. In addition, the team conducted further experiments [18] to compare the 3D-printed bionic lung heat exchanger with the microchannel heat exchanger. The air temperature on the hot side of the bionic lung heat exchanger decreased from 500 °C to 383.2 °C, and the air temperature on the cold side increased from 25 °C to 344.3 °C. The air temperature on the hot side of the microchannel radiator decreased from 500 °C to 367.9 °C, and the air temperature on the cold side increased from 25 °C to 330.4 °C, so the heat transfer efficiency of the 3D-printed bionic lung heat exchanger is higher than that of the traditional microchannel radiator. Pracht et al. [19] developed an aluminum 3D-printed heat exchanger with fins. The research showed that compared with traditional plate-fin heat exchangers, the designed geometry has advantages in the laminar flow range, with higher heat transfer efficiency and lower pressure drop. Compared with existing heat exchangers, the heat exchanger can achieve the same heat transfer capacity while reducing the volume by half. Efficiency improvement is obvious. Wang et al. [20] used additive manufacturing methods to prepare and test several alumina radiators to verify their actual performance. The results showed that this method can completely form the complex cooling structure inside the radiator, and has higher surface quality. The cooling hydraulic drop of the radiator was reduced by 19.8%, the thermal resistance was reduced by 11.8%, and the temperature distribution was more uniform. In summary, many researchers have carried out a lot of research on the performance improvement and application of 3D-print-based radiators, and the current research aims to apply 3D printing technology to traditional air-cooled radiators and water-cooled radiators to achieve lightweight and improve heat dissipation performance. However, there is still a lot of potential in the lightweightness of the 3D printing radiator, and the composite radiator based on 3D printing technology can further improve the performance of the radiator.

## 2. Experimental Setup and Simulation Model

### 2.1. Experimental Setup

#### 2.1.1. Test Parts

**Figure 1:**
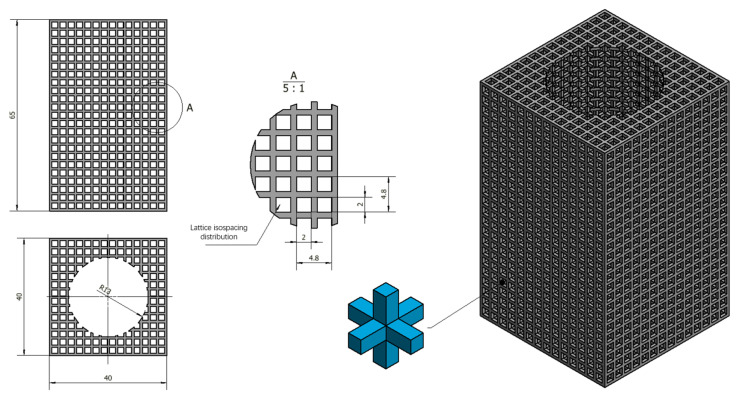
Lattice isospacing distribution.

The battery heat dissipation module lattice skeleton has external dimensions of 40 mm × 40 mm × 65 mm (L × W × H). The reserved hole size is 26 mm for the battery mounting holes. The lattice skeleton hole size is 2 mm × 2 mm × 2 mm (L × W × H), which are the length, width, and height of the three directions of equally spaced uniform distribution in the entity part of the final formation of the lattice skeleton features. The crystalline spore structure is shown in Figure 1. The cross-section size of the lattice spore is 0.8 mm × 0.8 mm. The lattice structure model is a kind of structure that repeats periodically in three-dimensional space and connects with itself uniformly and transitionally. It is very important to choose the appropriate cell type. The cell unit is used in this structural design, as shown in Figure 1. Inside the lattice cell, there are 6 struts of the same length and cross-section connecting the center of the body to the 6 vertices, and the periodic arrangement of the lattice cells finally constitutes a rectangular dot matrix structure. The main design parameters of the dot matrix structure include the length of the rectangular cell rods (s = 0.8 mm) and the length of the cell rods L (L = 1 mm). The cellular unit has a simple structure, isotropic characteristics, and strong self-supporting ability. It can be used as the unit of the grid structure, which can effectively improve the utilization of the material and the mechanical properties of the structure.

#### 2.1.2. Prototype Preparation of the Heat Exchanger

**Figure 2:**
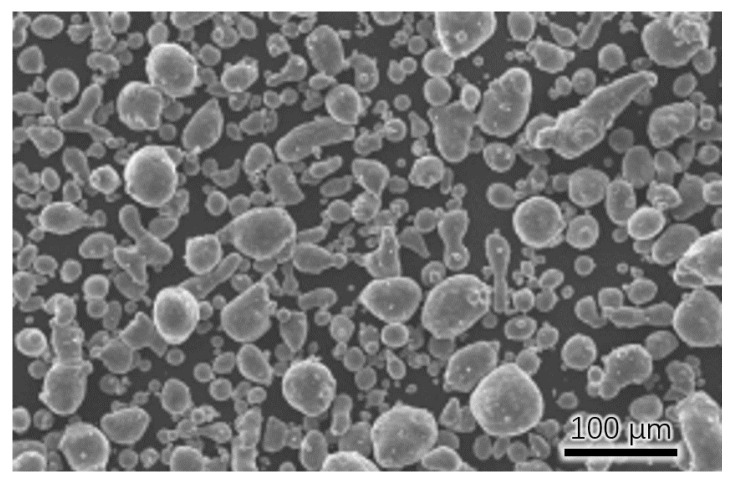
SEM topography of AlSi10Mg powder.

This lattice frame was printed using vacuum induction melting aerosolized AlSi10Mg aluminum alloy powder, which is the most widely used aluminum alloy material in the field of additive manufacturing, with good thermal and electrical conductivity, and other chemical compositions, as shown in Table 1. SEM topography of the AlSi10Mg powder is shown in Figure 2. The powder particles are spherical, the particle size range is 15–53 μm, the particle size distribution D50 is 32–38, the bulk density is 1.3 g/cm^3^, and the vibration density is 1.55 g/cm^3^.

**Figure 3:**
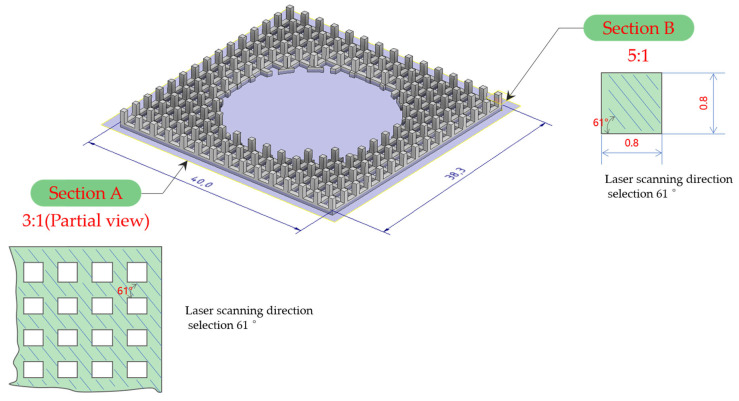
Schematic diagram of the lattice-structure-forming process.

Finite element analysis of the lattice-structure-forming process was carried out by using simufact additive software before forming to predict the dimensional accuracy of the forming as well as the possible curves. The lattice structure is formed using laser selective melting and formingequipment FS-271M (Hunan Huashu High tech Co., Ltd., China). The maximum laser output power of the equipment is 500 W, and the maximum size of the formed part is 275 mm × 2750 mm × 320 mm. The parameters of AlSi10Mg-forming are as follows: the laser power is 400 W, the scanning speed is 1100 mm/s, the thickness of the powder is 50 μm, and the scanning spacing is 110 μm. The scanning method of lattice scanning is line scanning, and there are two kinds of cross-sections in the scanning process of the lattice structure, which are as wide as 0.8 mm tic-tac-toe frame and 0.8 mm square, The single-layer laser scanning path is shown in Figure 3, in the first section of the internal scanning, and then scanning the border. In the next layer of scanning, the laser selects 61°, and so on back and forth. The column needs to be completed by laying 50 layers of powder, and the plane needs 16 layers. During the forming process, the most difficult forming area is the lower surface of the upper frame, which is the overhanging surface, but the overhang length is only 0.8 mm, which is not supported to reduce the post-processing work of the parts. The forming process uses nitrogen protection; oxygen content is less than 100 ppm; the forming substrate material is 6061 aluminum alloy; preheating temperature is 150 °C; afterwards, the parts are formed in the nitrogen protection of 300 °C insulation 2 h annealing treatment. The same batch of print material tensile specimens are placed horizontally on the substrate.

**Figure 4:**
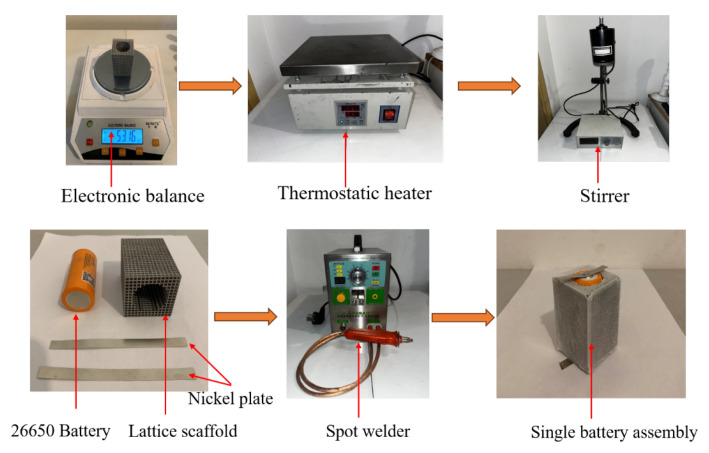
Assembly of the prototype of test sample.

After the 3D lattice structure was prepared, a 26650 Li-ion batterywas skillfully inserted into the structure and the voids of its frame were filled with PCM material to form a composite structure, as shown in Figure 4. In order to deeply investigate the effect of the introduction of the lattice frame on the heat transfer capability of the heat exchanger, the maximum temperature performance of the lithium 26650 battery in the heat exchanger equipped with the lattice frame and the pure PCM heat exchanger under the same conditions is compared, and the change of the liquid phase rate of the PCM is analyzed in both models. The preparation steps of the heat sink prototype are as follows: first, the required amount of bulk PCM was accurately weighed using an electronic scale and placed in a beaker. Subsequently, the PCM was heated using a thermostatic heater until it was completely melted. During this process, a high-speed stirrer was utilized to continuously stir to ensure that the solid and liquid portions of the PCM were evenly mixed. After the cells were securely placed in the lattice frame, a syringe was used to precisely fill each gap in the frame with liquefied paraffin. Next, the nickel wafers were finely welded by means of a spot welder in order to facilitate the connection to the charging and discharging devices in subsequent experiments. Finally, in order to monitor the temperature change of the battery in real time, we pasted a type K thermocouple on the surface of the battery.

#### 2.1.3. Geometry Detection

Laser scanner (K30, Zhongmei company, Hangzhou, China) was used to complete the three-dimensional scanning of the lattice solid features, Geomagic control X data geometry inspection software was used to import the digital standard model, and three-dimensional scanning of the slice data was used to detect the external dimensions of the lattice structure.

The use of the traditional three coordinates and scanning methods cannot be detected on the lattice size, so we used image recognition methods to detect the surface quality of the lattice parts. The lattice skeleton holes in the outer surface of the workpiece are the main index of the experimental detection. A graphic recognition detection system was established to detect the formation quality of a single outer surface of the lattice. Due to the small hole imaging principle of the camera, when the incident light is not perpendicular to the imaging plane, it is very easy to shoot the internal side walls of the skeleton hole, which results in a large error in the calculation of the area of the skeleton hole. Therefore, this experiment uses a linear CCD camera (precision), the camera is mounted on the X and Y direction guide rail, and the workpiece target is continuously photographed.

#### 2.1.4. Tensile and Compression Test

**Figure 5:**
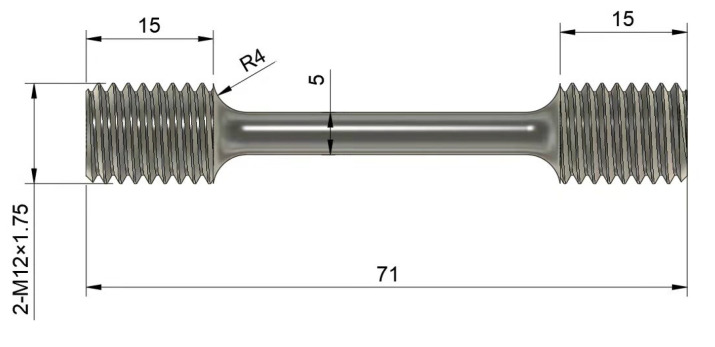
Tensile specimen size.

With the same heat treatment of the lattice structure through mechanical processing to obtain the standard tensile specimen as in Figure 5, on the testing machine for the standard tensile test, we measured the material tensile curve and fit it to the material model. The fitting data are used to predict the strength of the lattice structure. The lattice structure strength was tested by performing compression tests on the formed lattice structure under the same conditions.

The tensile and lattice structures were examined by microscope for low magnification. Low magnification corrosion solution was 10% NaOH, and room-temperature corrosion was conducted for 3 min. The parts were examined by microscope for high magnification; the corrosion solution was hydrofluoric acid: nitric acid: water = 1:3:5 diluted 20 times. The corrosion time was 7 s. Fracture detection was carried out using a scanning electron microscope.

#### 2.1.5. Heat Dissipation Evaluation

**Figure 6:**
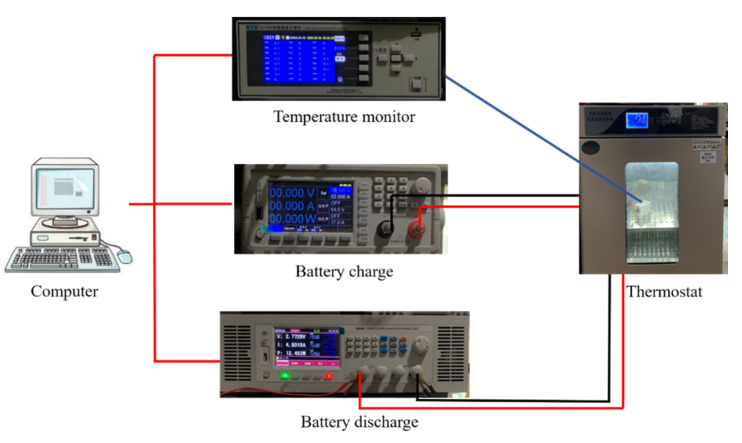
Experimental equipment and testing process.

The devices and equipment used in the experiment are shown in Figure 6. The battery charger model is HSP-6015, with an operating range of 0∼60.5 V, 0∼15.5 A. The battery is discharged by a constant current discharger (BEICH-CH9812, BEICH Electronic Technology Co., Ltd., China), with an operating range of 0∼150 V, 0∼120 A. The temperature of the battery can be adjusted from −100 to 1370 °C. The temperature monitor model is HZK-PZ-1024S, which is used to monitor the temperature of the battery, and its operating range is −100∼1370 °C. The thermostat model is HWS-50B (China), which can regulate the ambient temperature from 0∼60 °C. The simulation was performed using Fluent software (additive 2021).

### 2.2. Simulation Model

Fluent software was used for the simulation analysis. The initial temperature and ambient temperature of the simulation were set to 30 °C, the battery was discharged at a multiplication rate of 3 C, and the natural convection heat transfer coefficients of the battery and the lattice frame and the PCM in contact with the air were all set to 5 W/(m^2^·K). The thermophysical properties of the battery and the PCM are shown in Table 2. In order to ensure the simulation accuracy, the residual convergence criterion of the energy equation is set to 1 × $10^{-8}$.

## 3. Results and Discussion

### 3.1. Numerical Simulation of Forming Process

**Figure 7:**
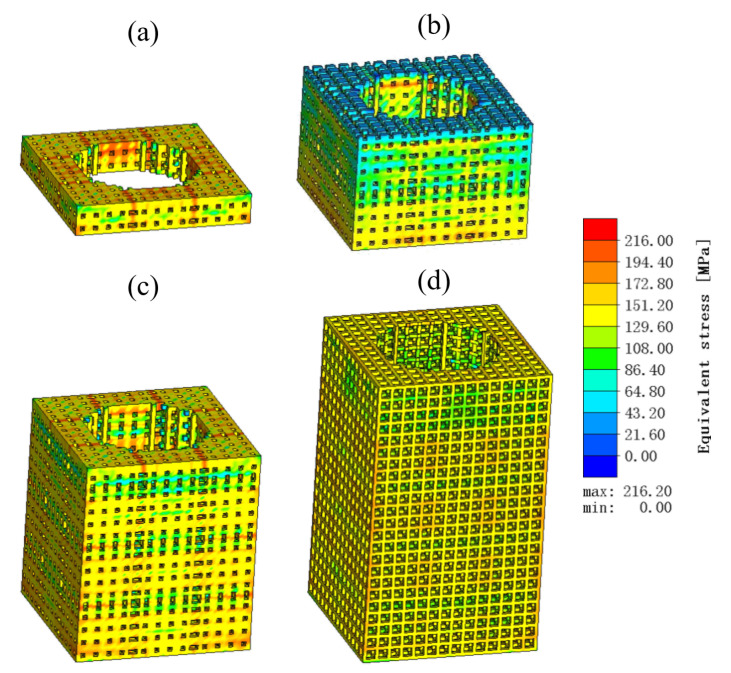
Lattice structure LPBF molding process equivalent stress field distribution. (**a**) Deform 10%. (**b**) Deform 40%. (**c**) Deform 70%. (**d**) Deform 100%.

The LPBF-forming process involves the rapid conversion of metal powder material to a multilayer solid–liquid–solid. Using the traditional thermoelastic–plastic finite element method to simulate the calculation is very difficult to realize. Simufact Additive uses the solid-state strain method to obtain the part LPBF-forming process of the stress–strain field, deformation, molding distribution, and other issues. In order to ensure that the lattice structure can not be eliminated by meshing in the simulation process, a 0.2 mm square hexahedral cell grid (pixel grid) is used to mesh the lattice parts, with a total of 136,429 meshes divided into 38 layers. The cloud diagram of equivalent stress distribution at different stages of the lattice structure LPBF molding process is shown in Figure 7, and the stress distribution of the part during the printing process changes continuously with the printing process. At the 10% stage of forming, the part structure is simple and uniformly distributed, so the equivalent stress is small; as the forming process continues, the lower part and the support region of the part are subjected to greater stresses, which may cause defects such as warping and support pull-off. As can be seen from the figure, the maximum equivalent stress value of the part is 216.2 Mpa. A larger stress will lead to a larger deformation of the part; the maximum equivalent stress of the part occurs in the four prongs of the part and the bottom region, while the part body stress distribution is relatively uniform.

**Figure 8:**
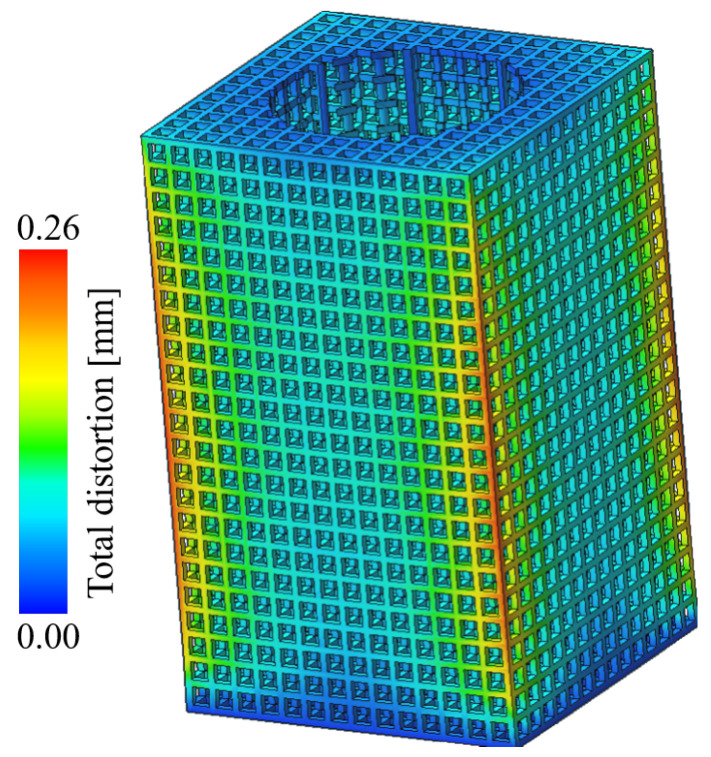
Lattice structure LPBF molding deformation prediction.

The deformation of the lattice structure after molding is shown in Figure 8. As can be seen from the figure, the body of the part is almost not deformed, and the largest deformation area occurs in the middle of the four prongs of the part, with a maximum deformation size of 0.25 mm. No molding defects were found in the simulation results.

### 3.2. Lattice Geometry and Metallographic Inspection

**Figure 9:**
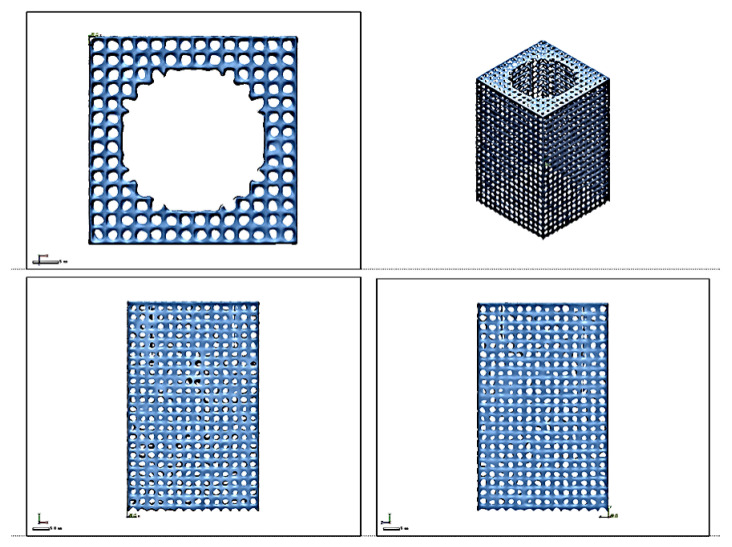
Lattice structure scanning results.

The lattice structure was scanned in three dimensions using the Hangzhou Zhongmei scanK30 laser scanner, and after fitting the triangular face sheet through the point cloud data, it was found that the end feature edges appeared to be partially missing in small microfeatures, as shown in Figure 9. This manifested as the end not being aligned, and the features did not follow the standard digital model with regular presentation, especially in the data fitting of the lattice holes. The holes were of different sizes, which ultimately led to the overall data accuracy of the larger deviation.

**Figure 10:**
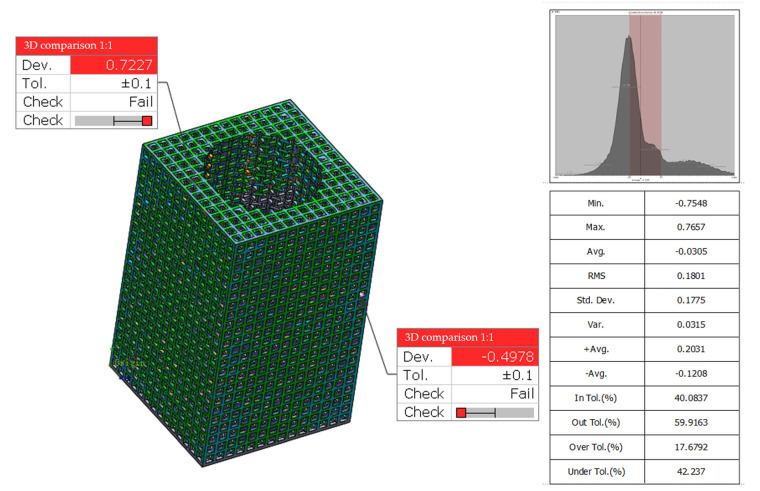
Analysis results of dimensional accuracy of lattice structure.

Using GeomagiccontrolX data geometry inspection software, the digital standard model was imported with the STL data of the slice after 3D scanning, and after preliminary alignment and reference point alignment, the data as a whole maintained a spatial coordinate system alignment relationship, as shown Figure 10. By comparing the 3D data and setting the standard deviation value to ±0.1, the final standard deviation value of the data is 0.1775, which meets the tolerance range of ±0.1 for 40% of the data. The discrete part of the data deviation occurs in the deformed part of the facet fit, and the point of occurrence of the maximum deviation value occurs at the end position of the facet fit. Therefore, the comparison and data analysis concluded that 3D data scanning of lattice microfabricated features is not the best choice.

### 3.3. Lattice Image Recognition Detection

**Figure 11:**
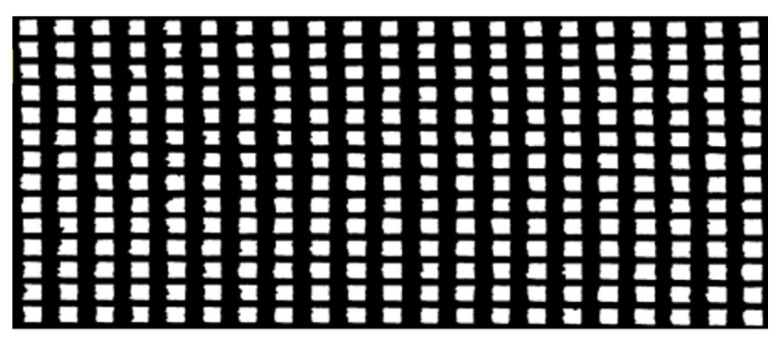
Image recognition results of physical samples.

**Figure 12:**
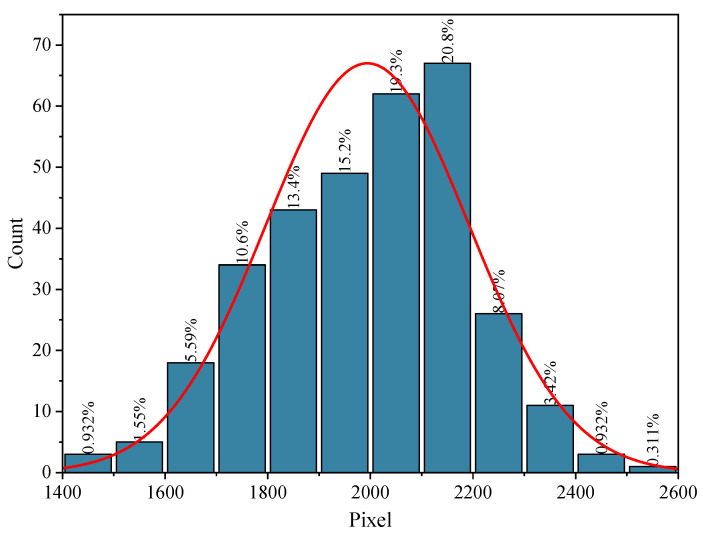
Relationship between the number of pixels and pores.

Graphical recognition technology was used to obtain the high-definition image of the side of the part, as shown in Figure 11. It shows the distribution of each area. The pixels of lattice part holes are between 1416 and 2504, with an average pixel number of 1994. The minimum size of the hole is NO.125, 1416 pixels; the largest is NO.275 and NO.2504 pixels. From the enlarged image, it can be seen that the two sides of the hole and the bottom surface of the hole form better; the size of the hole does not have much impact. Through image data processing, it was found that the distribution of pore size in LPBF formed materials follows a normal distribution, and the relationship between the number of pores and their pixels is shown in Figure 12. It affects the main area for the upper surface. Due to the overhanging surface, the hole of the upper surface is of poor quality, directly affecting the quality of the part molding.

### 3.4. Metallographic Inspection

**Figure 13:**
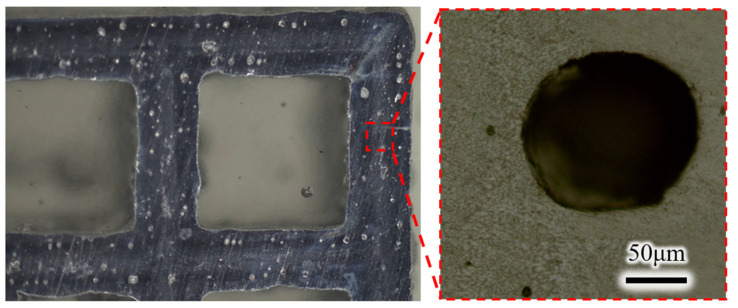
Low-magnification observation chart.

The mesh border size uniformity is basically 0.8 mm, the border cross-section design is for 0.8 mm square, and the actual print deformation is about an 0.8 mm diameter circle, which may be related to the equipment printing accuracy, etc. In Figure 13, it can be seen that for the border cross-section of the larger size of the holes, the molding quality is relatively low, but for the non-structural parts used for heat dissipation, instead, it is good, and the large number of holes can increase the heat dissipation area. Metallographic polishing and corrosion or the specimen (Figure 13) can be clearly seen parallel to the printing direction. For the melt pool buildup morphology, the melt pool depth is basically 50 μm or so. For the thickness of the laying of powder perpendicular to the printing direction, with the melt pool width of 110 μm or so, the laser and scanning spacing is the same. In addition to this, a large number of holes can be seen, but, on the contrary, this is conducive to the dissipation of heat.

### 3.5. Tensile and Compression Test

**Figure 14:**
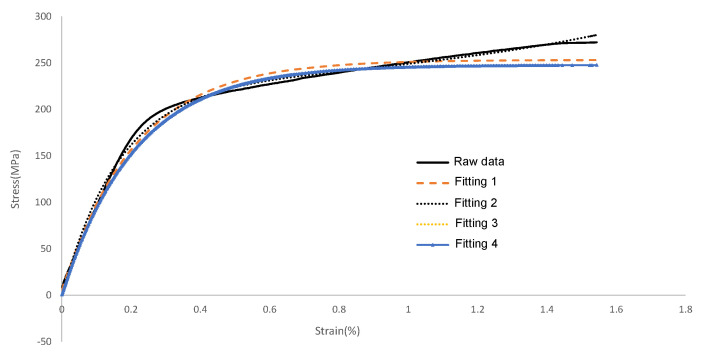
LPBF molding AlSi10Mg room-temperature tensile curve.

The tensile specimens after annealing were examined, and the tensile strength of LPBF-formed AlSi10Mg was 323 MPa, the yield strength was 217 MPa, and the elongation after break was 10.8%; its tensile curve is shown in Figure 14. Based on the characteristics of the raw data of the tensile curve of LPBF-formed AlSi10Mg, we designed four curve fits based on exponential functions. The different fitting models and parameters are shown in Table 3. The results show that the model with serial number 2 is in the best agreement with the original data.

Its fitting Equation (1) is as follows:

(1) $$y=9.504e^{1.223x}-228.2e^{-6.065x}+217.3$$

It has the highest goodness of fit with R^2^ of 0.9964, error sum of squares SSE of 7495, standard deviation RMSE of 4.78, and average relative error of 6.68%.

### 3.6. Point Structure Compression Test Analysis

**Figure 15:**
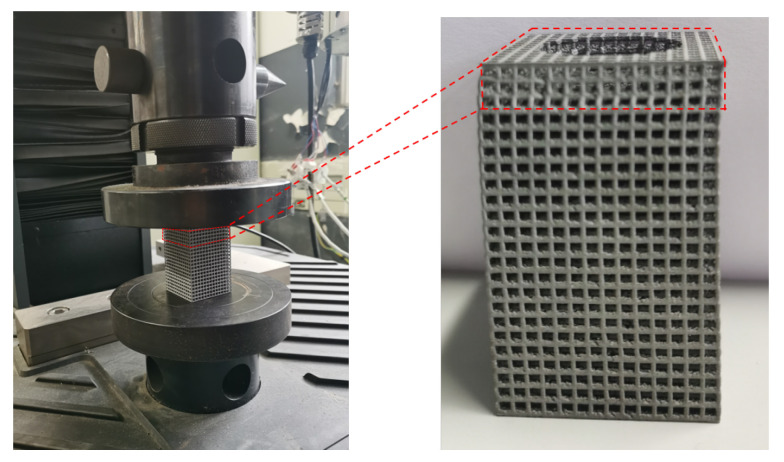
Compression experiments and local deformation of specimens.

**Figure 16:**
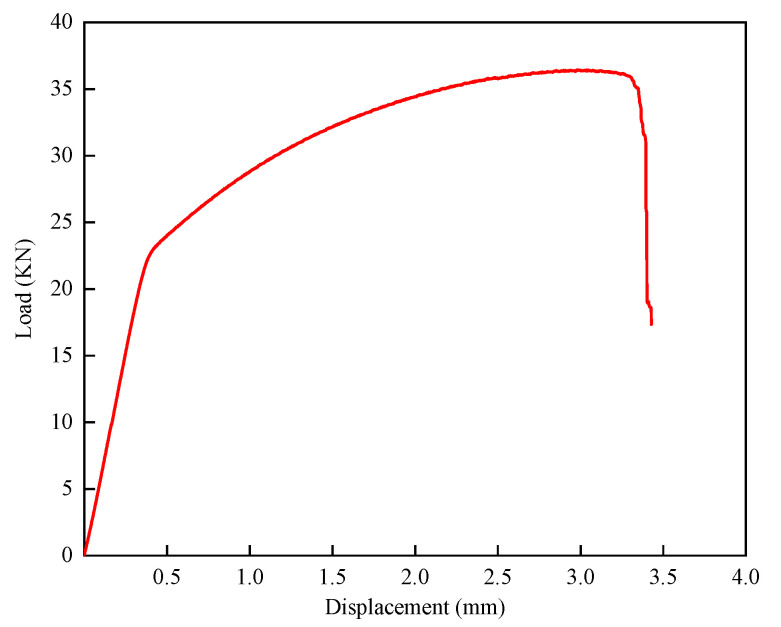
Diagram of the relationship between lattice compression load and displacement.

Compared with the uniform deformation of the mesh structure obtained from the simulation analysis, only the top four layers of the mesh structure were significantly deformed in the actual compression process, as shown in Figure 15. During the deformation process, the relationship curve between load and displacement is shown in Figure 16.

### 3.7. Heat Dissipation Performance

**Figure 17:**
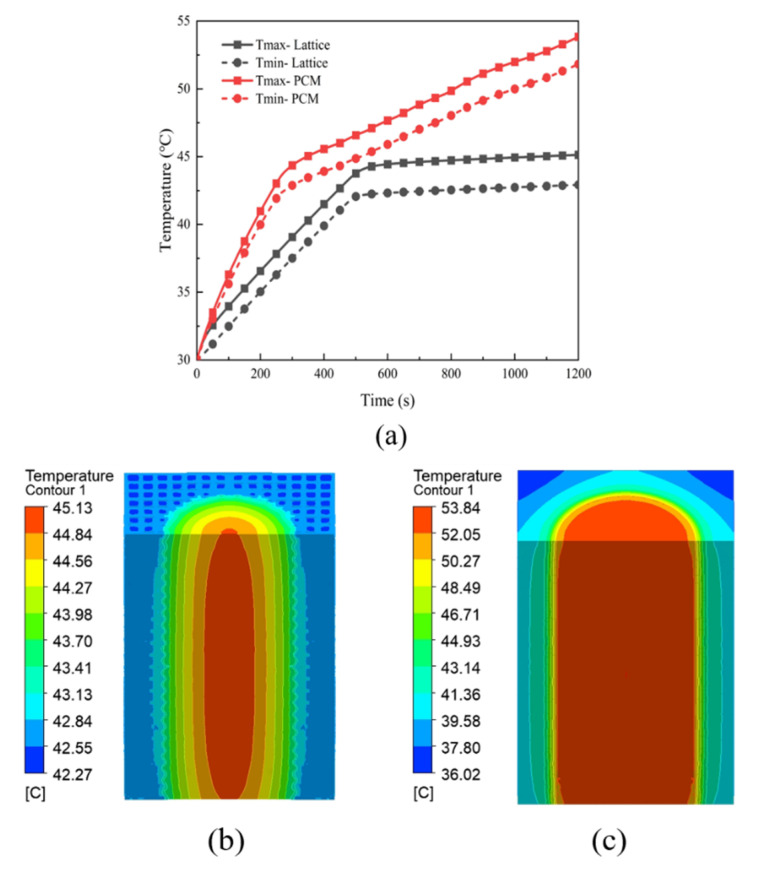
Maximum temperature curve and temperature cloud of the battery under two kinds of heat exchangers. (**a**) Plot of Li-ion battery temperature versus time. (**b**) Temperature cloud of cell profile under lattice heat exchanger. (**c**) Plot of cell profile temperature under pure PCM heat exchanger.

From Figure 17a, it can be seen that the maximum and minimum temperatures of 26650 Li-ion battery under the pure PCM heat exchanger are 55.84 °C and 51.82 °C, respectively, which are much higher than those under the lattice heat exchanger (45.13 °C and 42.92 °C), and the slope of the temperature curve of the battery under the pure PCM heat exchanger is steeper, which indicates that the battery’s temperature rises more rapidly, and there is an inflection point of the battery temperature curve within the interval of 200 s∼300 s, after which the slope of the curve slows down. There is an inflection point in the battery temperature curve in the interval of 200 s∼300 s, and the slope of the curve slows down after the inflection point. The reason for this inflection point is that before the inflection point, the temperature of the battery has not yet reached the melting point of the PCM, so the temperature will rise rapidly, and after the battery temperature reaches the melting point, the PCM starts to melt, and in the process, it will absorb a lot of the heat generated by the battery, so the temperature of the battery rises at a slower rate. The lattice heat exchanger battery temperature in the 500 s before the same also shows a rapid rise, but the melting point of the PCM after the battery temperature basically maintains stability, unlike the pure PCM heat exchanger, which continues to rise. This is because the addition of the lattice framework greatly improves the thermal conductivity of the PCM, so that the heat is generated by the battery in the heat exchanger more quickly, and the PCM far away from the battery is also able to absorb the heat.

Figure 17b,c show the profile temperature clouds of the battery under the lattice heat exchanger and pure PCM heat exchanger, respectively, which indicates that the high-temperature area of the battery with the lattice heat exchanger is significantly smaller than that of the pure PCM heat exchanger, and the temperature of the lattice heat exchanger is more uniform, whereas the pure PCM heat exchanger has an obvious temperature gradient that decreases from the inside to the outside. In addition, the lowest temperature at the edge of the lattice heat exchanger is 42.27 °C, while the lowest temperature at the edge of the pure PCM heat exchanger is 36.02 °C, which also shows that the lattice framework substantially improves the thermal conductivity of the PCM, so that the heat can be transferred faster and more uniformly in the heat exchanger; this improves the utilization rate of the PCM.

**Figure 18:**
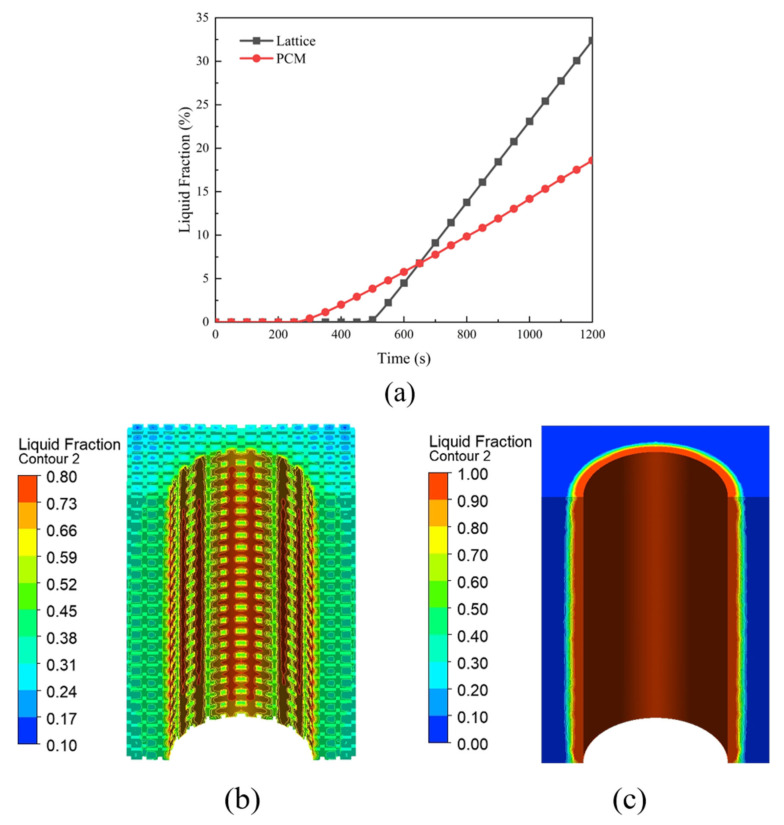
Liquid phase rate profiles and cloud plots of PCM under two heat exchangers. (**a**) Plot of cell discharge time versus average liquid phase rate. (**b**) Cloud plot of liquid phase rate for the profile of lattice heat exchanger. (**c**) Cloud plot of liquid phase rate for the profile of pure PCM heat exchanger.

In Figure 18a, it can be seen that the average liquid phase rate of the PCM with the lattice heat exchanger is higher at the end of cell discharge, reaching 32.4%, while the liquid phase rate of the PCM with the pure PCM heat exchanger is only 18.6%. The PCM in the lattice heat exchanger also starts to undergo phase change later, which is because the lattice framework enhances the PCM thermal conductivity and reduces the accumulation of heat in the PCM close to the cell surface, and the pure PCM heat exchanger does not allow the heat to be transferred to the PCM farther away efficiently due to the low thermal conductivity, so the PCM close to the cell surface will start to melt earlier. Figure 18b,c show the liquid phase rate cloud diagrams of the lattice heat exchanger and the pure PCM heat exchanger, respectively, from which it can be clearly seen that the lattice framework enhances the heat transfer capability of the PCM. The melting range of the pure PCM heat exchanger mainly concentrates near the surface of the battery because the battery constantly generates heat and is limited by the low thermal conductivity of the PCM, so it causes all the PCMs near the surface of the battery to melt, but the PCMs a little farther away from the surface of the cell start to melt earlier. The PCM near the battery surface melts all the time, but the PCM a little farther away does not undergo phase change, which does not take advantage of the large latent heat of the PCM, resulting in a waste of resources. On the other hand, the PCM in the lattice heat exchanger melts more uniformly; almost all of them are in a semimolten state, which can maximize the ability of the latent heat of the PCM.

**Figure 19:**
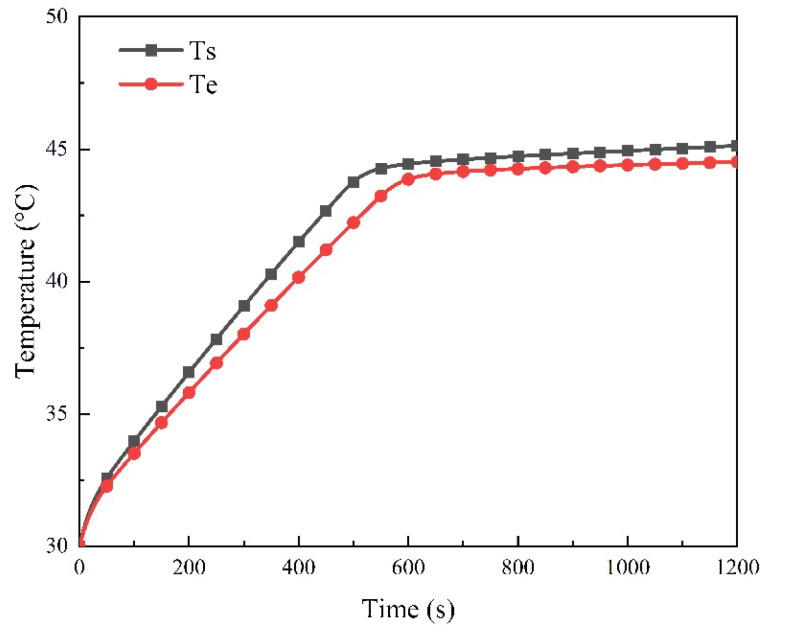
Comparison of experimental and simulation results.

From Figure 19, it can be observed that the melting time of PCM in the experiment is slightly later than that in the simulation, which may be due to the uneven gap between the battery and the heat sink, thereby increasing the contact thermal resistance. In addition, the simplification of the battery heat production model and the variation of internal resistance with temperature may also lead to the deviation. However, at the end of the experiment, the maximum temperature of the battery obtained from the experiment is basically consistent with the simulation results, and the maximum deviation is only 1.4%, indicating that the simulation results are reliable.

In summary, it can be seen from the simulation as well as the experimental results that the addition of the lattice framework is very significant in improving the heat exchanger performance. Compared with the pure PCM heat exchanger, the maximum temperature of the cell decreases by 16.2% after adding the lattice framework, and the average liquid phase rate of the PCM increases by 74.2%.

## 4. Conclusions

Through modeling, simulation, and analysis of the lattice structure of the battery heat sink module, as well as the subsequent comprehensive study of LPBF-forming and mechanical and corrosion-resistant experiments, we draw the following important conclusions:

- The introduction of the lattice skeleton significantly improves the thermal conductivity of the PCM. This improvement enables the heat generated by the cell in the heat exchanger to be transferred more rapidly, and the part of the cell far away from the phase change material can also absorb the heat effectively, thus realizing the efficient distribution of heat in the system.
- The lattice skeleton not only accelerates the heat transfer rate, but also makes the heat distribution in the heat exchanger more uniform. This uniformity not only improves the utilization rate of the PCM and reduces the waste of energy, but also helps to maintain the stability of the cell temperature, which in turn prolongs the service life of the cell.
- The melting process of PCM is more uniform in the lattice heat exchanger. This feature maximizes the latent heat capacity of PCM and makes it more useful in the battery heat dissipation process.

In summary, by optimizing the grid structure of the battery heat dissipation module and introducing the lattice skeleton, we successfully improved the performance of the heat dissipation system, which provides a strong guarantee for the efficient and stable operation of the battery. This research result not only provides a new idea for the development of battery heat dissipation technology, but it also provides a useful reference for the application in related fields.

## Figures and Tables

**Table 1:** Chemical composition of AlSi10Mg powder.

Si	Mg	Zn	Cu	Fe	Ni	Mn	Al
10.24	0.21	0.01	0.056	0.09	0.015	0.036	Bal.

**Table 2:** Physical and thermophysical parameters of battery and paraffin parts.

Battery Parameters	Values	Paraffin Parameters	Values
Height/mm	65	Density/kg·m^−3^	810
Diameter/mm	26	Specific heat capacity/J·(kg·K)^−1^	2000
Capacity/Ah	4.5	Thermal conductivity/W·(m·K)^−1^	0.2
Specific heat capacity/J·(kg·K)^−1^	1108	Latent heat/kg·(m·s)^−1^	275
Thermal conductivity/W·(m·K)^−1^	3.91	Viscosity/kg·(m·s)^−1^	0.0035

**Table 3:** LPBF-forming AlSi10Mg material tensile curve fitting.

NO.	Fitting Model	a	b	c	d	f	SSE	R^2^
1	a(1 − $e^{bx}$) + c	247.8	−4.764	5.415	/	/	22,780	0.9891
2	a($e^{bx}$) + c($e^{dx}$) + f	9.504	1.223	−228.2	−6.065	217.3	7495	0.9964
3	a/(1 + $e^{bx}$) + c	468.9	−7.25	−221.5	/	/	34,960	0.9833
4	a + b(1 − $e^{-cx}$) + d(1 − $e^{-fx}$)	13,140	247.8	−4.764	−13,140	−43,980	22,783	0.9891

## Data Availability

The data presented in this study are available on request from the corresponding author.
